# High-Density Distributed Crack Tip Sensing System Using Dense Ultra-Short FBG Sensors

**DOI:** 10.3390/s19071702

**Published:** 2019-04-10

**Authors:** Xin Gui, Zhengying Li, Xuelei Fu, Changjia Wang, Yiming Wang, Hongli Li, Honghai Wang

**Affiliations:** 1 School of Information Engineering, Wuhan University of Technology, Wuhan 430070, China; guixin@whut.edu.cn (X.G.); xlfu@whut.edu.cn (X.F.); wangchangjia@whut.edu.cn (C.W.); wangyiming@whut.edu.cn (Y.W.); lihl@whut.edu.cn (H.L.); 2 National Engineering Laboratory for Fiber Optic Sensing Technology, Wuhan University of Technology, Wuhan 430070, China; wanghh@whut.edu.cn

**Keywords:** optical fiber sensing, crack tip, dense ultra-short fiber Bragg grating, high density, distributed sensing

## Abstract

Crack generation starts at the crack tip, which bears the highest stress concentration. Under further stress, the crack propagates and leads to severe structural damage. To avoid such damage, the identification of the crack tips, and monitoring of the surrounding stress and strain fields, are very important. In this work, the location of, and strain distribution monitoring around, the crack tip are achieved using a dense ultra-short (DUS) fiber Bragg grating (FBG) array together with an improved optical frequency domain reflectometry (OFDR) interrogator. The adjacent grating interference correlation algorithm helps overcome the limitation on the demodulation precision, which is imposed by the inherently broad reflection spectra of individual ultra-short gratings. High spatial resolution measurement of the strain profile around the crack tip is performed at different levels of induced strain. Furthermore, the vertical-crossed layout is adopted to avoid the omission of cracks, which usually occurs in the case of the one direction layout. We achieve 1 mm spatial resolution and 7.5 m detection distance. Location of a single crack, multiple cracks, and an oblique crack was realized experimentally by locating the crack tips. The experimental results are consistent with the theoretical analysis, verifying the feasibility of the DUS-FBG system for high-density distributed crack tip sensing.

## 1. Introduction

Local cracks are a typical form of structure damage caused by stress concentration [1]. As an ideal sensing technique for embedded measurement and damage detection in micro-structures, optical fiber sensors (OFS) possess some unique features, e.g., small size, flexibility, distributed measurement and immunity to electromagnetic interference [2,3,4,5]. Compared to fiber-based sensing technology using intrinsic scattering (Raman, Brillouin and Rayleigh), fiber Bragg grating (FBGs) -based technology can achieve higher signal-to-noise-ratios (SNRs), since it possesses much higher back-scattering light coupling efficiency [6], while FBG-based technology is more suitable for high-prision crack strain detection. Takeda et al. have used a variety of FBG sensors to perform a series of studies on different form of damage. By embedding a small-diameter FBG sensor into the carbon fiber reinforced plastic (CFPR) laminates, delamination in the CFPR crossply laminates can be detected [7]. The embedding of chirped FBG (CFBG) into the composite, on the other hand, leads to the location of the crack. To monitor the tip location of a crack, the strain state of an interface crack is memorized by the plastic deformation of a metal wire before being picked up by two FBG sensors [8]. By comparing the variation of the experimentally measured spectrum with the simulated results, the crack length in the fatigue crack propagation is monitored [9,10,11].

However, these techniques only use multiple FBG sensors to detect a number of key points, exhibiting difficulty in distributed crack detection. Complex non-homogeneous strain distributions arise when cracks are present [12]. Strain measurements and position identification of the crack tip can be realized with optical frequency-domain reflectometry (OFDR) technology. Based on the high-resolution and high-accuracy measurement of longitudinal strain distribution, crack detection is facilitated [13]. Non-uniform strain measurement with 1 mm spatial resolution near the crack tip is feasible by using multiplexed weak FBG sensors [13] or long-length FBG sensors [14] based on OFDR. However, the limited detection range of this method restricts its application to the monitoring of small-scale crack distribution. Meanwhile, the conventional layout of FBG is only in one direction. Such arrangement cannot determine the angle between the crack and the sensor placement, which is not capable of predicting the development trend of crack tip propagation [15].

In FBG-based high-density distributed crack tip sensing, the effective length of an individual sensor determines the crack detection range and the FBG layout density. To achieve a large measurement range with high precision, long detection distance and large multiplexing capacity need to be maintained [16,17,18,19,20]. For a typical FBG sensor array, the multiplexing capacity is limited by the spectral shadowing and the multiple-reflection crosstalk effects [21]. By reducing the single FBG reflectivity, the crosstalk effects can be suppressed, and multiplexing of 2000 ultra-weak FBGs over a single fiber is realized [22]. Recently, we demonstrated the use of a dense ultra-short (DUS) FBG array for distributed sensing [23,24]. Combined with the OFDR technology, the large multiplexing capacity of the DUS-FBG array facilitated long detection distance distributed sensing of 10 m, with a high spatial resolution of 1.5 mm. Even though the capability of long-distance detection with high spatial resolution is ideal, the limited demodulation accuracy, imposed by the inherent wide spectral width of individual FBGs, hinders the application of such a system for distributed crack tip sensing [25,26].

In this work, we propose to use a DUS-FBG array and an improved OFDR interrogator for distributed crack tip sensing. Measurement with high spatial resolution is guaranteed by the ultra-short length and spacing of each grating. In addition, we have overcome the precision issue by using the interference spectrum of the FBG formed by two adjacent gratings, instead of the reflection spectrum of a single FBG. Wavelength demodulation can thus be achieved with significantly improved precision. Strain profile measurement is carried out for different levels of induced strain, which identifies the locations of crack tips. Instead of the commonly used one direction layout, we adopt a vertical-crossed layout of FBGs, which effectively eliminates the issue of crack omission. High spatial resolution of 1 mm and long detection distance of 7.5 m are achieved. Experimental results are consistent with the theoretical analysis, verifying the practical advantages of small size, light weight, multiplexing capability and resistance the system’s feasibility for the high-density distributed crack tip sensing system.

## 2. Experimental Setup and Principle

### 2.1. Distributed Sensing System

**Figure 1:**
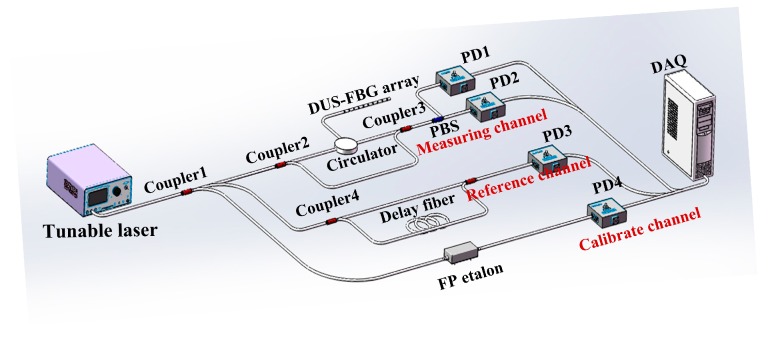
Improved OFDR demodulation system: PBS (Polarization Beam splitter); PD (Photodetector); DAQ card (Data Acquisition Card).

Figure 1 shows the schematic of the distributed OFS system with the improved OFDR interrogator. Light from a tunable laser is split into three channels: the measuring channel, the reference channel and the calibration channel. The optical signals from the three paths are converted into electrical signals by three photodetectors (PDs) and acquired synchronously by a data acquisition card (DAQ). The measuring channel is a Mach-Zehnder interferometer (MZI) with polarization diversity detection. The DUS-FBG array in one arm of the MZI is later applied for distributed crack tip sensing. The polarization diversity technique is used to mitigate the signal fading due to misalignment of the reflected signal and the local-oscillator fields [27]. While the structures of the reference and calibration channels are the same as those in the DUS-FBG array based OFDR sensing system [28], signals obtained from the measuring channel are treated differently. The FBG writing system adopts the phase mask method, in which a line-narrowed ArF excimer laser (OptoSystmes CL5300, Optosystems Ltd., Moscow, Russia) with a beam size of 4 mm × 12 mm, pulse width of 10 ns and maximum pulse energy of 40 mJ was used in the FBG writing platform. The whole FBG fabrication system is controlled and synchronized by a computer via the interface circuits. With this system, the large-scale multiplexed FBGs can be made continuously, and the FBG parameters can be controlled in a certain range. This on-line large-scale production system not only eliminates the splicing loss but also produces FBGs with more precisely controlled parameters.

### 2.2. Adjacent Grating Interfernce Correlation Algorithm

**Figure 2:**
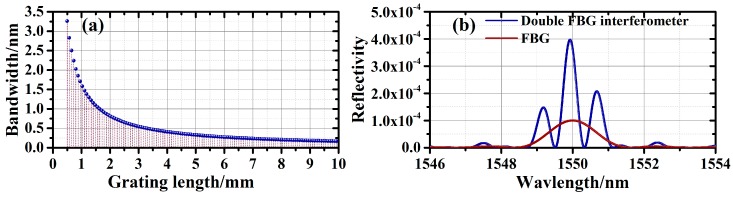
(**a**) Relationship between the grating length and the bandwidth of the reflection spectrum; (**b**) simulated single-FBG reflection spectrum and double-FBG interference reflection spectrum.

Conventionally, the Gaussian fitting algorithm and cross-correlation algorithm can be used to perform wavelength demodulation in the OFDR interrogator. These demodulation techniques rely on the narrow reflection spectra of FBGs to achieve high demodulation accuracy. However, to facilitate high spatial resolution and large-scale multiplexing of the gratings, the length of the FBGs considered in this work is kept ultra-short. As shown in Figure 2a, the bandwidth of the FBG reflection spectrum is thus inherently broad, which is unfavorable for wavelength demodulation. To overcome this issue, we adopt the double-FBG interferometry with the adjacent grating interference correlation algorithm for the demodulation. Figure 2b shows the simulated single-FBG reflection spectrum and double-FBG interference spectrum. The two gratings, both having lengths of 0.5 mm and reflectivity of 40 dB, are separated by 0.5 mm. Based on double-FBG interferometry, the sensing information can be obtained by tracking the overall movement of the interference spectrum. With the information of the interference spectrum at a reference temperature/strain level, the relative wavelength shift can be calculated by a cross-correlation algorithm, which can then be converted into a relative change in the temperature/strain level. Consequently, measurement of the absolute temperature/strain is realized. Compared to the single-FBG reflection spectrum, the interference spectrum has a much narrower peak, which leads to higher demodulation accuracy.

The beat signal containing sensing information from the measuring channel is resampled by using frequency multiplying algorithm [29]. Each two adjacent FBG are treated as one interferometer. The time and frequency pair in fast Fourier transform (FFT) corresponds to the wavenumber and position. By sliding a rectangular bandpass filter, amplitude distribution along the frequency (position) direction in the frequency domain of the double-FBG interference spectrum can be obtained. Inverse fast Fourier transform (IFFT) is then applied to obtain the beat frequency as a function of optical wavelength for the double-FBG interferometer. Then, the window slides with the prescribed distance for the same process, until the whole wavenumber range is covered. In this way, the reflected spectrum at an arbitrary position of each adjacent double-FBG can be obtained, and the sensing distribution can be calculated by the cross-correlation algorithm.

**Figure 3:**
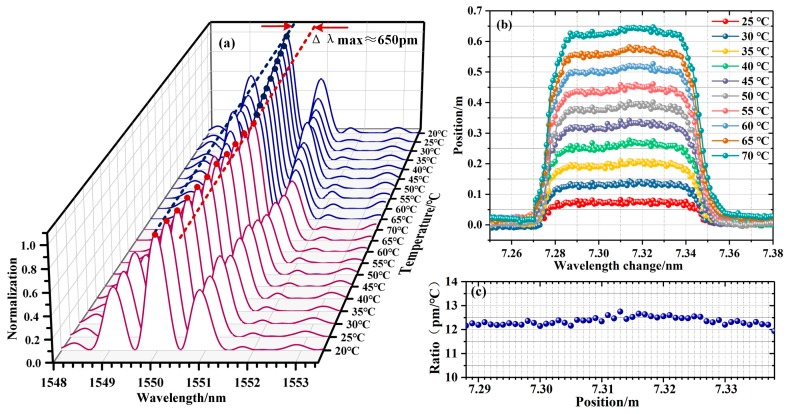
The distributed temperature sensing: (**a**) the reflection spectrum changes of one sensing element area in different temperature; (**b**) the wavelength changes with the temperature change; (**c**) the temperature sensitivity of each element in FBG array sensor.

To verify the improved demodulation accuracy based on the interferometry technique, we conducted a temperature sensing experiment based on a 5 cm DUS-FBG array. The grating length and spacing are kept at 0.5 mm, and the reflectivity is kept at −45 dB. With a multiplexing capacity of 7500, the total length of the DUS-FBG array is 7.5 m. Test sensor array was written on a single-mode fiber which was pulled by 40 g tension to move at 15 m/min, the laser ran at 167.67 Hz for 30 s, with 10 ns pulse width and 8 mJ pulse energy. The temperature is varied from 20 °C to 70 °C, with a step of 5 °C. The relative wavelength shift is obtained using the double-FBG interferometry cross-correlation algorithm. Each two adjacent gratings form a sensing element. In the following, a sensing element is selected for analysis. Figure 3a shows the double-FBG interference spectrum change at different temperatures. Data obtained at 20 °C is chosen as the reference for the Double-FBG interferometry cross-correlation algorithm. The 0.05 m region from 7.287 m to 7.338 m is temperature controlled. As shown in Figure 3b, the wavelength shifts in the heated region are nearly 650 pm for a temperature change of 50 °C. Figure 3c shows the sensitivities of each sensing elements which is estimated with linear curve fitting and ranges from 11.92 pm/°C to 12.66 pm/°C, with an average temperature sensitivity of 12.34 pm/°C.

**Figure 4:**
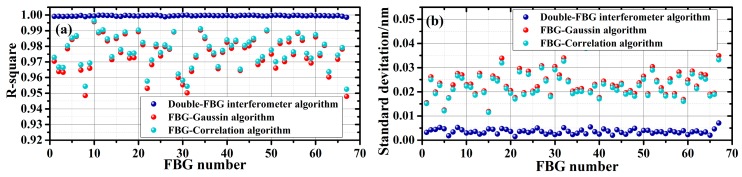
Comparison of the three techniques with distributed temperature sensing: (**a**) linearity of temperature sensing characterized by the R-square value; (**b**) wavelength demodulation accuracy.

In addition to the cross-correlation algorithm, the double-FBG interferometry and the Gaussian fitting algorithm are also using to obtain and analysis the sensing data. Calibration of the temperature sensitivity is performed by linear fitting, and we obtained the standard deviation (STD) values of each measuring point by averaging 20 measurements. Comparison of the three demodulation techniques are plotted in Figure 4. The blue, red, and green dots correspond to the results obtained from the double-FBG interferometry, the Gaussian fitting algorithm, and the cross-correlation algorithm. As shown in Figure 4a, the double-FBG interferometry exhibits best linearity, exhibiting a mean R-square value of 0.999. With the Gaussian fitting algorithm and the cross-correlation algorithm, the mean R-square values decrease to 0.974 and 0.976, indicating degraded linearity. Figure 4b shows the distribution accuracy of the three demodulation techniques. The average wavelength demodulation accuracy for the three cases are 3.57 pm, 23.73 pm and 22.73 pm, respectively. A higher demodulation accuracy is clearly obtained based on the double-FBG interferometry method.

## 3. Crack Tip Position Detection

**Figure 5:**
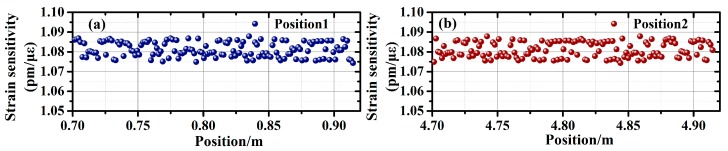
Comparison of the three techniques with distributed strain sensing: (**a**) linearity of strain sensing characterized by the R-square value; (**b**) wavelength demodulation accuracy.

Crack tips lie where the strain gradient is the maximum. Identification of a crack tip can be achieved by measuring the strain profile. With the high spatial resolution provided by double-FBG interferometry, the DUS-FBG in this work can combine with the OFDR technique to perform strain profile monitoring. Strain calibration is then applied to the same DUS-FBG sensor array using the proposed double-FBG interferometry. Two 0.22 m sections, located at 0.70 m to 0.92 m and 4.70 m to 4.92 m, are stressed using a translation stage. The strain is varied from 33.33 με to 366.63 με, at a step of 33.33 με. Figure 5a,b shows the strain sensitivity coefficients, which are obtained at the two sections, respectively. Both sections show an average strain sensitivity of 1.08 pm/με. The average measurement errors of the wavelength shifts are 17.88 pm and 17.92 pm for the two sections, corresponding to strain measurement accuracies of 16.55 με and 16.59 με.

### 3.1. Single Crack Detection

We first investigated the identification of a single crack. Four aluminum tensile specimens, with width of 16 cm, thickness of 1 cm, and crack lengths of 3 mm, 6 mm, 9 mm, and 12 mm, were prepared. A crack tip was created by applying a load to the specimen. To compare with the experimental results, a finite element (FE) model of the crack tensile specimen was constructed by ANSYS, which analyzed the influence of crack length and load on the strain field at the crack tip. A DUS-FBG sensor array composed of 7500 gratings was applied for the experimental demonstration. The gratings were 500 μm in length and separated by less than 500 μm. Tensile loading testing was performed using an electronic universal material testing machine (3300, Instron. Inc., Norwood, MA, USA, maximum tensile loading of 100 kN) which applied tensile loading along the FBG sensor direction. The top boundary was fixed and a uniform tensile loading is applied to the bottom. For each crack length, 10 different levels of force, from 1 kN to 10 kN, with a step of 1 kN, were applied to the aluminum specimens. At each load level, the strain profile is obtained.

**Figure 6:**
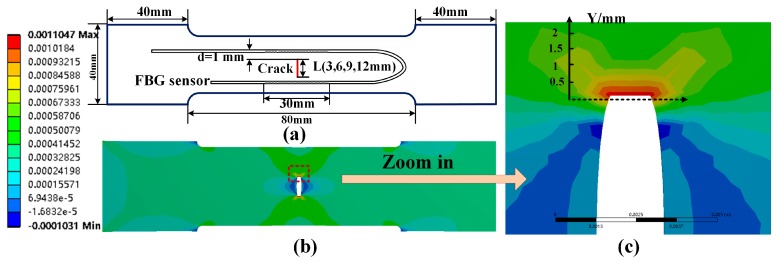
The experiment of loads with different length crack: (**a**) sensor placement on Specimen 1; (**b**) simulated distributed strain profile surrounding a 9 mm crack and a load level of 5 kN; (**c**) zoom-in view of the strain profile around the crack tip.

**Figure 7:**
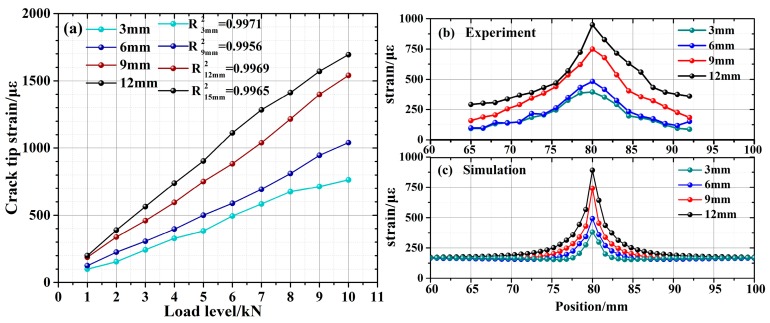
Strain measurement at different crack lengths and load levels: (**a**) experimentally measured crack tip strain; (**b**) experimentally measured non-uniform strain distribution; (**c**) simulated non-uniform strain distribution around the crack tip.

From the simulation in Figure 6b,c, it was observed that the strain concentration area at the crack tip is within 1.5 mm perpendicular to the crack. The sensors were glued uniformly on the specimen surface using liquid cyanoacrylate adhesive, and were placed perpendicular to the crack with 1 mm distance from the crack tip. Figure 7a shows that the strain at the crack tip increases with the applied stress, as expected. In addition, longer crack length resulted in higher strain, indicating a higher possibility of crack expansion. As shown in Figure 7b, the experimentally obtained strain profiles were able to identify the location where the strain gradient is the maximum, which corresponded to the crack tip. For comparison, the strain profile at the sensor position was simulated and plotted in Figure 7c. It was observed that the experimental results were in good agreement with the simulation results. The discrepancy between the simulation and the actual detection were attributed to the slight error of the arrangement of the sensor. In addition, the pre-crack was affected by the processing technology, which caused the crack width of the actual experimental specimen to be slightly wider than the design, and also resulted in the profile difference between experiment and simulation.

### 3.2. Multiple Crack Detection

**Figure 8:**
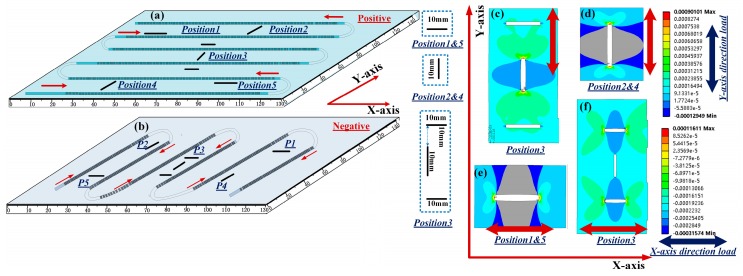
Sensor placement on (**a**) the front side and (**b**) the back side of Specimen2. Simulated strain distributed at (**c**) Position 3 and (**d**) Position 2 & 4 when the external force is applied along the *Y*-axis direction with 5 kN load. Simulated strain distributed at (**e**) Position 1 & 5 and (**f**) Position 3 when the external force is applied along the *X*-axis direction with 5 kN load.

Conventionally, the one direction layout is applied for crack tip detection. Such an arrangement would lead to omission of the cracks that are parallel to the FBG sensors. To solve this problem, we adopted a vertical-crossed layout, in which the FBGs placed on one side of the specimen, parallel to each other, were perpendicular to the FBGs on the other side. The feasibility of this layout is verified by presenting cracks that are in two perpendicular directions on the aluminum specimen. All pre-cracks considered here had 1 cm length and 1 mm width. Figure 8a,b shows the positions of the pre-cracks and the layout of the DUS-FBG arrays on the specimen. The two 750 mm sensor arrays were composed of thousands ultra-weak FBGs with 0.5-mm grating length and grating spacing, around 50 dB reflectivity, and 1549.85 nm center-wavelength at room temperature. The FBG array in the front was perpendicular to that at the back one. To facilitate efficient crack tip detection, the DUS-FBGs were arranged so that they were 1 mm away from the cracks. The transverse tensile force and the longitudinal tensile force were applied to the specimen respectively, external load from 0 to 5 kN, with a step of 1 kN is applied to the specimen. Figure 8c–f is the simulated strain distributions at different crack positions when an external load of 5 kN was considered.

**Figure 9:**
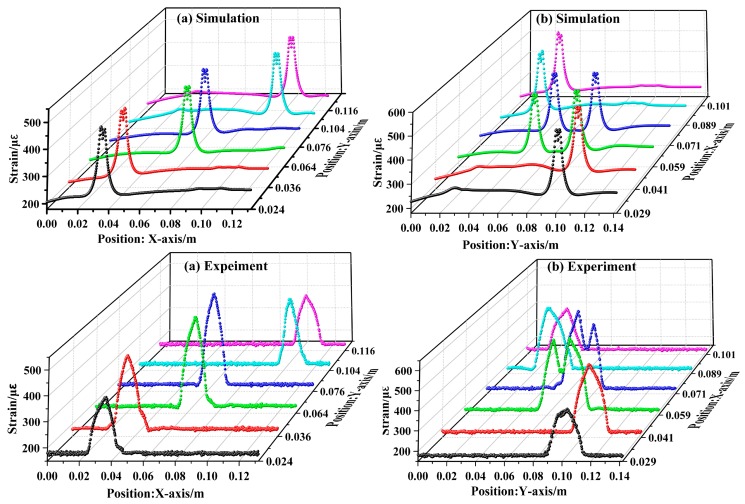
Multiple–crack sensing with 5 kN load applied along (**a**) the *x*-axis and (**b**) the *y*-axis.

The strain profiles of the cracks at different positions in two direction loads were then measured using the DUS-FBG arrays. Figure 9a shows the simulated strain distribution and the experimentally measured results, when the external force is applied along the x-axis direction with 5 kN load, while Figure 9b is obtained when the same amount of force is applied along the *y*-axis direction. The experimental results are in good agreement with the simulation results in Figure 8a and Figure 9c. The simulation was carried out in strict accordance with the position perpendicular to the 1-mm of the crack. However, during the layout of the FBG array, the manual operation inevitably led to some errors. Since the strain varied as the distance between the crack and the sensor changes, the measurement accuracy was slightly affected by the positioning errors. This analysis shows that the DUS-FBG array sensor could be used as a tool to locate crack tip position. Also, distributed crack monitoring is more reliable with the vertical-crossed layout, which presents no uncertainty in the crack location and avoids the omission of parallel cracks.

### 3.3. Inclined Crack Detection

**Figure 10:**
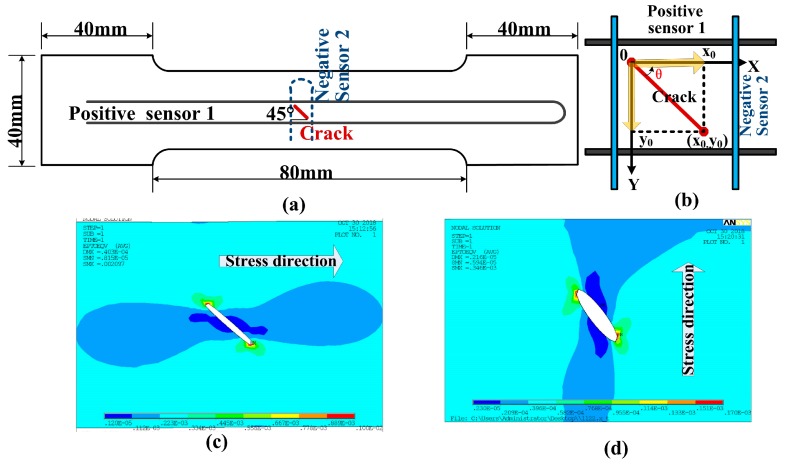
(**a**) Sensor placement in specimen3; (**b**) schematic diagram of the method for determining the crack angle; strain distributed simulation: strain distributed simulation: (**c**) *X*-axis load; (**d**) *Y*-axis load.

**Figure 11:**
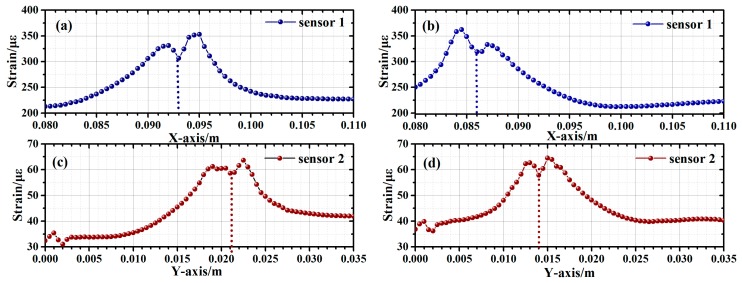
Inclined crack specimen non-uniform strain on *X*-axis 5 kN load: (**a**) Sensor1 Position 1; (**b**) Sensor1 Position 2; *Y*-axis 5 kN load: (**c**) Sensor2 Position 1; (**d**) Sensor2 Position 2.

In addition, the vertical-crossed layout can also facilitate position and distributed strain sensing of inclined cracks. Figure 10a shows the schematic of 1-cm thick aluminum Specimen 3, on which a 45° incline crack is created and two FBG sensor arrays were placed using the vertical-crossed layout. As depicted by Figure 10b, the two sensors were placed 1-mm away from two crack tips, respectively. External force was then applied along the *x*-axis and the *y*-axis. As shown in Figure 10c,d, strain was mainly generated close to the two crack tips, along the direction of the external force. Therefore, location of the incline crack could be realized by the location of the two crack tips on the *xy*-plane. For crack tip location, we defined the position of one crack tip as the origin, as shown in Figure 10b. Strain distribution along the sensor array is simulated to verify the feasibility of the proposed technique. The external forces were applied along the two orthogonal directions, both induced by 5 kN load. Figure 11a,b presents the strain at the two crack tips, when the external force was applied along the *x*-axis. The high-strain regions coarsely located the two crack tips, while the dip in the center corresponds to the exact position. The coordinate positions of the two crack tips on the *x*-axis were thus found to be 93 mm and 86 mm, respectively. Figure 11c,d shows the results obtained with the external force applied along the *y*-axis. The corresponding coordinate positions of the two crack tips on the *y*-axis were 21 mm and 14 mm, respectively. With these two positions identified, the crack length and angle were calculated to be 9.90 mm and 45°, according to the Pythagorean Theorem. A simulation was also performed on two specimens with 10 mm cracks of 15° and 30° incline angles. The average errors of the crack length and angle were 1.40% and 2.10%, respectively.

**Figure 12:**
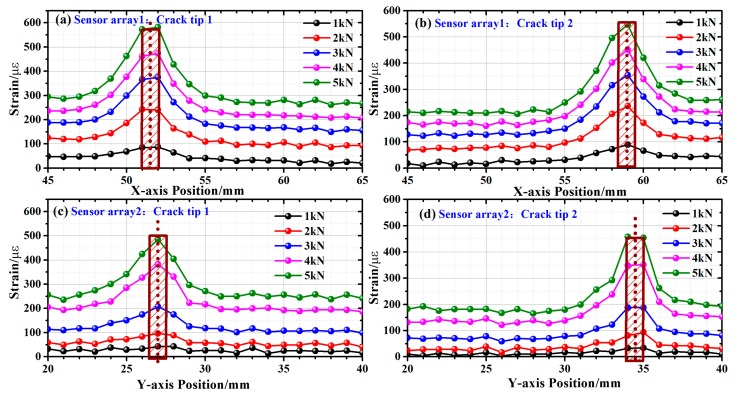
Inclined-crack sensing with 1kN-5-kN load applied along (**a**,**b**) the *x*-axis; (**c**,**d**) the *y*-axis.

We then carried out the incline crack sensing experiment on three specimens with 10-mm cracks of 15°, 30°, and 45° incline angles. Figure 12 shows the results obtained with the 45° specimen at different load levels from 1 kN to 5 kN, at a 1 kN step. Even though the induced strain was stronger with higher load, location of the crack tips could be achieved at all load levels, since the maximum strain gradient occurs at the same position in all five cases. The calculated crack length was 10.63 mm and the angle were 48.81°, exhibiting 6.30% and 8.48% errors, respectively. Considering the results obtained with the 15° and 30° specimens, the average errors were 7.35% and 9.15% in the experiment. The larger errors in the experiment were attributed to the insufficient spatial resolution, and the position of the crack tip could only be identified with 1 mm accuracy. Meanwhile, the manual installation of the sensor arrays inevitably led to positioning errors, which affected the accuracy of the strain sensing results. Therefore, in the future work, in order to improve the accuracy of crack length and angle acquisition, we need to further improve the spatial resolution of the distributed sensing system.

## 4. Conclusions

DUS-FBG arrays, combined with the improved OFDR interrogation method, are well suited to the measurement of distributed strain around the crack tips, and thus, to locating cracks. The commonly used transfer matrix method fails to predict the multiple peaks due to the highly non-uniform strains in the vicinity of cracks. The OFDR-based technique is capable of directly characterizing the local strain state along an FBG sensor without additional information collected from other sources (e.g., FE modeling). The analysis of the crack tip position, length and angle can all be achieved by the DUS-FBG based vertical-crossed layout, without omission of the cracks that are parallel to the FBG sensors. This kind of low reflectivity DUS-FBG with short length and extremely small spacing can easily be fabricated. In addition, the increase of bandwidth can slow down the intensity decay rate to a certain extent without affecting the crosstalk level, leading to increased multiplexing capacity. The accuracy of measurements reported in the paper is also confirmed by the good agreement between the experimental and the FE simulation results. With the double-FBG interferometry, the improved OFDR system has sufficiently high spatial resolution to identify and interrogate each one of the closely spaced ultra-short FBG sensors. Distributed demodulation with 1-mm spatial resolution and 7500 multiplexing capacity was achieved. In practice, the accuracy of the strain sensing is affected by the experimental environment (especially temperature), and further improvement in the performance can be realized by temperature compensation methods, such as adding a reference grating or using polarization-maintaining fiber grating for strain sensing. The next step for this study will be to compare classical variables, such as crack path, crack growth life.

## References

[1] Takeda S., Minakuchi S., Okabe Y., Takeda N. (2005). Delamination monitoring of laminated composites subjected to low-velocity impact using small-diameter FBG sensors. Compos. Part A Appl. Sci. Manuf..

[2] Tsuda H., Lee J.R., Guan Y., Takatsubo J. (2007). Investigation of fatigue crack in stainless steel using a mobile fiber Bragg grating ultrasonic sensor. Opt. Fiber Technol..

[3] Takeda S.I., Aoki Y., Nagao Y. (2012). Damage monitoring of CFRP stiffened panels under compressive load using FBG sensors. Compos. Struct..

[4] Hu H., Li S., Wang J., Wang Y., Zu L. (2016). FBG-based real-time evaluation of transverse cracking in cross-ply laminates. Compos. Struct..

[5] Zhang W., Zhang M., Wang X., Zhao Y., Jin B., Dai W. (2019). The Analysis of FBG Central Wavelength Variation with Crack Propagation Based on a Self-Adaptive Multi-Peak Detection Algorithm. Sensors.

[6] Ding Z., Wang C., Liu K., Jiang J., Pan G., Pu Z., Liu T. (2018). Distributed optical fiber sensors based on optical frequency domain reflectometry: A review. Sensors.

[7] Takeda S., Yamamoto T., Okabe Y., Takeda N. (2007). Debonding monitoring of composite repair patches using embedded small-diameter FBG sensors. Smart Mater. Struct..

[8] Minakuchi S., Yamauchi I., Takeda N., Hirose Y. (2012). Memorizing and detecting an arrested crack in a foam-core sandwich structure using embedded plastic materials and fiber-optic sensors. Smart Mater. Struct..

[9] Jin B., Zhang W., Ren F., Zhang M., Dai W., Wang Y. (2017). Mechanism of Subordinate Peak Skewing of FBG Sensor during Cracks Propagation Monitoring on Aluminum Alloy Structure. J. Sens..

[10] Jin B., Zhang W., Zhang M., Ren F., Dai W., Wang Y. (2017). Investigation on characteristic variation of the FBG spectrum with crack propagation in aluminum plate structures. Materials.

[11] Kakei A., Epaarachchi J.A., Islam M., Leng J. (2018). Evaluation of delamination crack tip in woven fibre glass reinforced polymer composite using FBG sensor spectra and thermo-elastic response. Measurement.

[12] Colpo F., Humbert L., Botsis J. (2007). An experimental numerical study of the response of a long fibre Bragg grating sensor near a crack tip. Smart Mater. Struct..

[13] Ning X., Murayama H., Kageyama K., Wada D., Kanai M., Ohsawa I. (2014). Dynamic strain distribution measurement and crack detection of an adhesive-bonded single-lap joint under cyclic loading using embedded FBG. Smart Mater. Struct..

[14] Sans D., Stutz S., Renart J., Mayugo J.A., Botsis J. (2012). Crack tip identification with long FBG sensors in mixed-mode delamination. Comopos. Struct..

[15] Belotteau J., Berdin C., Forest S., Parrot A., Prioul C. (2009). Mechanical behavior and crack tip plasticity of a strain aging sensitive steel. Mater. Sci. Eng. A.

[16] Chan C.C., Jin W., Wang D.N., Demokan M.S. (2003). Intrinsic crosstalk analysis of a serial TDM FGB sensor array by using a tunable laser. Microw. Opt. Technol. Lett..

[17] Soller B.J., Gifford D.K., Wolfe M.S., Froggatt M.E. (2005). High resolution optical frequency domain reflectometry for characterization of components and assemblies. Opt. Express.

[18] Ou Y., Zhou C., Qian L., Fan D., Cheng C., Guo H. (2015). Large-capacity multiplexing of near-identical weak fiber Bragg gratings using frequency-shifted interferometry. Opt. Express.

[19] Zhou L., Li Z., Xiang N., Bao X. (2018). High-speed demodulation of weak fiber Bragg gratings based on microwave photonics and chromatic dispersion. Opt. Lett..

[20] Han P., Li Z., Chen L., Bao X. (2017). A High-Speed Distributed Ultra-Weak FBG Sensing System with High Resolution. IEEE Photonics Technol. Lett..

[21] Wang Y., Gong J., Dong B., Wang D.Y., Shillig T.J., Wang A. (2012). A large serial time-division multiplexed fiber bragg grating sensor network. J. Lightwave Technol..

[22] Luo Z., Wen H., Guo H., Yang M. (2013). A time- and wavelength-division multiplexing sensor network with ultra-weak fiber Bragg gratings. Opt. Express.

[23] Xin G., Li Z., Fu X., Wang C., Wang H., Wang F., Bao X. (2018). Large-scale multiplexing of a FBG array with randomly varied characteristic parameters for distributed sensing. Opt. Lett..

[24] Li Z., Tong Y., Fu X., Wang J., Guo Q., Yu H., Bao X. (2018). Simultaneous distributed static and dynamic sensing based on ultra-short fiber Bragg gratings. Opt Express.

[25] Cheng R., Xia L., Sima C., Ran Y., Rohollahnejad J., Zhou J., Wen Y., Yu C. (2016). Ultra-short FBG based distributed sensing using shifted optical Gaussian filters and microwave-network analysis. Opt. Express.

[26] Rui C., Li X. (2016). Interrogation of weak bragg grating sensors based on dual-wavelength differential detection. Opt. Lett..

[27] Song J., Li W., Lu P., Xu Y., Chen L., Bao X. (2014). Long-Range High Spatial Resolution Distributed Temperature and Strain Sensing Based on Optical Frequency-Domain Reflectometry. IEEE Photonics Technol. Lett..

[28] Gui X., Li Z., Wang F., Wang Y., Wang C., Zeng S., Yu H. (2017). Distributed sensing technology of high-spatial resolution based on dense ultra-short FBG array with large multiplexing capacity. Opt. Express.

[29] Xiang N., Li Z., Gui X., Wang F., Hou Y., Wang H. (2018). Research on a high-precision calibration method for tunable lasers. Meas. Sci. Technol..

